# CARDIORESPIRATORY FITNESS IS ASSOCIATED WITH CIRCADIAN BEHAVIORAL PARAMETERS OF PHYSICAL ACTIVITY IN OLDER ADULTS

**DOI:** 10.1093/geroni/igad104.1257

**Published:** 2023-12-21

**Authors:** Melissa Erickson, Terri Blackwell, Theresa Mau, Samaneh Farsijani, Lauren Sparks, Nancy Glynn, Steven Cummings, Karyn Esser

**Affiliations:** AdventHealth, Orlando, Florida, United States; California Pacific Medical Center, San Francisco, California, United States; San Francisco Coordinating Center, San Francisco, California, United States; University of Pittsburgh, Pittsburgh, Pennsylvania, United States; AdventHealth, Orlando, Florida, United States; University of Pittsburgh School of Public Health, Pittsburgh, Pennsylvania, United States; California Pacific Medical Center Research Institute, San Francisco, California, United States; University of Florida, Gainesville, Florida, United States

## Abstract

Cardiorespiratory fitness (VO2peak) declines with age, and this may be due in part to weakened circadian functions. Rhythmic patterns of rest-activity behavior over 24h represents a novel feature of physical activity, indicative of circadian behavior. Whether VO2peak relates to rest-activity rhythms (RAR) is unknown. We determined cross-sectional associations between VO2peak and RAR in SOMMA (N=714, Age: ≥70 yrs). Activity data from wrist-worn accelerometry (ActiGraph GT9X) were collected in one-minute epochs, over 8, 24h periods. An extension to the traditional cosine model was used to map RAR to activity data, calculating these parameters: strength of the rhythm (amplitude); robustness of rhythm (pseudo-F statistic); and timing of peak activity (acrophase). RAR parameters were expressed as quartiles. Linear models examining associations of RAR and VO2peak were adjusted for potential confounders (age, race, height, comorbidities and lifestyle). Interactions of RAR predictors with sex were examined. Associations between VO2peak and rhythmic amplitude appears to differ by sex (p-interaction=0.07). Among men, VO2peak was positively associated with amplitude (adjusted mean Q1: 21.3 vs. Q4: 23.9, p-trend=0.005)-even after adjusting for moderate-to-vigorous physical activity levels (MVPA)-although associations were more modest and not significant in women. Those with higher VO2peak had an earlier acrophase (time of peak activity) (adjusted mean: Q1 <1:30PM: 21.0 vs. Q4>3:09PM 19.3, p-trend <0.0001) and no sex interaction was observed. There were no associations between the robustness of RAR and VO2peak. Analyses reveal that better cardiorespiratory fitness relates to features of circadian behavior independent of MVPA, supporting emerging research that circadian-based interventions promote healthy aging.

